# Impact of Octenyl Succinylation and Bee Products on Maize Starch Films and Apple Storage Quality

**DOI:** 10.3390/ijms262311270

**Published:** 2025-11-21

**Authors:** Paulina Pająk, Karolina Królikowska, Lesław Juszczak, Gohar Khachatryan, Jacek Grzyb

**Affiliations:** 1 Department of Food Analysis and Evaluation of Food Quality, Faculty of Food Technology, University of Agriculture Balicka Str. 122, 30-149 Krakow, Poland; 2 Department of Dietetics and Food Studies, Faculty of Science & Technology, Jan Dlugosz University in Czestochowa, 42-200 Czestochowa, Poland; 3 Department of Microbiology and Biomonitoring, Faculty of Agriculture and Economics, University of Agriculture Balicka Str. 122, 30-149 Krakow, Poland

**Keywords:** maize starch, octenyl succinylation, film, honey-bee extracts, water barrier properties, thermal properties, antioxidant activity, antimicrobial effect, apple storage

## Abstract

Starch is a promising biodegradable polymer for food packaging, offering a competitive alternative to synthetic films, but its native form has limited functionality. This study aimed to develop edible films based on native (N) and octenyl-succinylated (OS) maize starch incorporating honey-bee-derived extracts (HBE), and to evaluate their physicochemical, structural, and bioactive properties. Moreover, the films were applied as a packaging for apple slices stored for seven days. OS starch enhanced film functionality, particularly when combined with bee pollen, bee bread, and propolis extracts. The presence of amphiphilic octenyl groups and bioactive components significantly modified film microstructure and thermal behavior. Compared to native starch films, OS-based films showed higher water solubility and swelling ratio but lower tensile strength. Among the HBE formulations, propolis-enriched films exhibited the highest total phenolic content, strongest antioxidant capacity, and most effective antimicrobial action. Although none of the starch-based films prevented apple weight loss or browning during storage, propolis addition markedly reduced mold growth compared to synthetic packaging. Overall, octenyl-succinylated maize starch combined with propolis extract offers a promising, biodegradable alternative to conventional plastic films for sustainable food packaging applications.

## 1. Introduction

The most commonly used materials that come into direct contact with food include paper, glass, metal, and petroleum-based plastics. The latter, due to their advantageous properties—such as a low production cost, lightweight nature, water resistance, and customizable flexibility or stiffness—are among the most widely used packaging types. Most plastics are single-use, not biodegradable materials; some of them are films intended for indirect contact with food. Their excessive production and use lead to environmental degradation, loss of aquatic life, and negative impacts on both animal and human health [1]. Current EU regulations concerning single-use petroleum-based packaging have compelled scientists and manufacturers to search for new, eco-friendly alternatives based on polymers from renewable sources [2]. Nowadays, intensive efforts have been made to develop packaging based on polysaccharides and proteins, which come into direct contact with the packaged product while being safe and fully edible, thus leaving no waste behind. Moreover, next-generation packaging should be designed to biodegrade easily while also enhancing food protection through, e.g., antioxidant and/or antimicrobial activity [3,4].

In a commercial sense, edible packaging, including coatings and films, refers to thin, food-grade polymer layers that can be safely consumed along with the products. Technically, an edible coating is a liquid polymer solution or dispersion that is applied directly to the surface of food by dipping, spraying, or panning (for example, wax on citrus fruits). In contrast, an edible film is a performed thin layer or solid sheet that can be used as a wrapping, covering, packaging material, or food separator (for example, thin films used as separators between cheese slices) [5].

Starch is one of the natural polymers, widely available and biodegradable. Its production is inexpensive, which makes it a promising component for producing edible films. Native starches form weak, cohesive, and rubbery pastes when heated and cooled; therefore, the manufacturers generally prefer modified starches with tailored functional properties [6]. The characteristics of starch can be improved through various modifications, including esterification with octenyl succinic anhydride. The resulting product, octenyl succinate starch (OS starch), acquires surface-active properties due to the presence of hydrophobic alkenyl groups and is commonly used as an emulsion stabilizer and texturizing agent and exhibits improved encapsulation capacity for bioactive compounds [6,7].

Currently, research is being conducted on the enrichment of food and packages with natural ingredients that potentially have food-preserving effects. As a result, active antioxidant packaging materials are most commonly developed by incorporating plant extracts, natural phenolic compounds, as well as vitamins C and E, and carotenoids [3,4]. Due to their unique structural characteristics, phenolic compounds display a range of physicochemical behaviors, such as binding to biological macromolecules (such as polysaccharides, proteins and lipids), as well as reacting with smaller entities (such as metal ions and free radicals). Moreover, they can form hydrogen bonds and engage in hydrophobic interactions with starch molecules, which can modify the starch paste’s thermal properties, pasting behavior, rheology, and gel structure to varying extents. As a result, phenolic compounds hold promise for enhancing the quality and functionality of starch-based films [4,8].

The bee’s by-products, such as honey, bee bread, pollen, and propolis, are excellent natural substances known for their health-promoting effects. They are rich in saccharides, organic acids, amino acids, polyphenolic compounds, flavonoids, carotenoid-like substances, Maillard reaction products, enzymes, waxes, and minerals [9,10]. These polar and non-polar compounds can pose challenges when preparing film-forming solutions based on native starch. Therefore, the use of esterified starch with amphiphilic OS groups can enhance emulsifying capacity and encapsulation efficiency for bee product extracts [11,12]. Although the physicochemical properties of starch-based films enriched with polyphenolic compounds have been reviewed in numerous reports [4,13,14,15,16], there is still a knowledge gap regarding how the properties of such films change when esterified starch is used and when HBE is incorporated. In our earlier studies [11,12] we presented pioneering research on the impact of selected bee products on OS films produced from potato starch. The incorporation of bee product extracts influenced the hydrophobicity, crystallinity, and optical and sensory qualities of OS potato films. However, recent studies demonstrated that films made from maize and potato starches differ in their physicochemical properties due to their botanical origin and variations in starch structure. Potato and maize starches differ in granule size, amylose-to-amylopectin ratio, and crystallinity. Potato starch granules are larger and richer in amylopectin, leading to different gelatinization, crystallinity, and film-forming properties compared to maize starch, which has smaller granules and a higher proportion of amylose. These structural differences influence their functional behavior in food and material applications [17]. For example, depending on the source cited, the tensile strength of films based on maize starch ranges from 1.4 to 7.1 MPa, whereas potato starch films exhibit better mechanical properties, ranging from 4 to 16 MPa. Elongation at break varies between 22.5% and 79.2% for maize starch, and between 12.6% and 93.5% for potato starch films. In terms of moisture-related properties, water solubility ranges from 13.5% to 25.64% for maize starch films, and from 6% to 28% for those based on potato starch. Furthermore, according to the literature, maize starch films generally show better water barrier properties compared to potato starch films, with water vapor permeability values of 17.7 × 10^−11^ for maize starch and between 5.6 and 84 × 10^−10^ for potato starch films. Moreover, the addition of bioactive compounds to the film-forming solution during film preparation influences the films’ properties in different ways [18,19,20,21].

Fresh-cut fruits are ready-to-eat products that are gaining increasing attention in the food market. However, peeling and cutting processes accelerate the metabolic activity of plant tissues, making these products more susceptible to spoilage compared to whole fruits [5,22]. The commercial success of fresh-cut fruits, such as apples, is strongly affected by color changes resulting from enzymatic reactions involving phenolic compounds. Therefore, it is essential to apply preservation techniques such as storage at low temperatures, modified atmosphere packaging, and, more recently, the innovative approaches like edible films enriched with antimicrobial and antioxidant agents. These strategies help extend the shelf life of fresh-cut fruits by reducing respiration rate, surface contamination, and tissue browning [5,22,23].

Considering that the botanical origin of starch may strongly influence the film properties and, potentially, also its ability to interact with HBE, studies on OS maize starch films are crucial for fully understanding the underlying interactions and for developing promising packaging materials. Thus, the aim of this study is to prepare edible films based on native and octenyl-succinylated maize starch, compare their physicochemical properties, and evaluate the quality of fresh-cut apples stored in these films for 7 days. It is hypothesized that the use of octenyl-succinylated maize starch in starch–HBE formulations will enhance the functional and bioactive properties of the resulting films, thereby improving the storage quality of the fruits.

## 2. Results and Discussion

### 2.1. Quantitative Determination of the Effectiveness of the Starch Esterification Process

Maize starch esterified with an octenyl succinic anhydride level of 7% was subjected to DS analysis. Determined degree of substitution was 0.013 ± 0.001. The obtained result proved that during starch esterification with OSA (octenyl succinic anhydride), the hydroxyl groups of the anhydroglucose units in starch are substituted with carboxyl groups of octenyl succinate. Similar results to those presented in the current study were reported by Lopez-Silva et al. [24], who employed a lower concentration of the modifying agent while extending the reaction duration. The DS value was recalculated as a percentage, and it was 0.78% ± 0.02. The content of octenyl succinate groups in the starch was below 3%, meeting the requirements for starch approval for use in the food industry [25].

### 2.2. Fourier Transform Infrared Spectroscopy with Attenuated Total Reflectance (FTIR-ATR)

**Figure 1:**
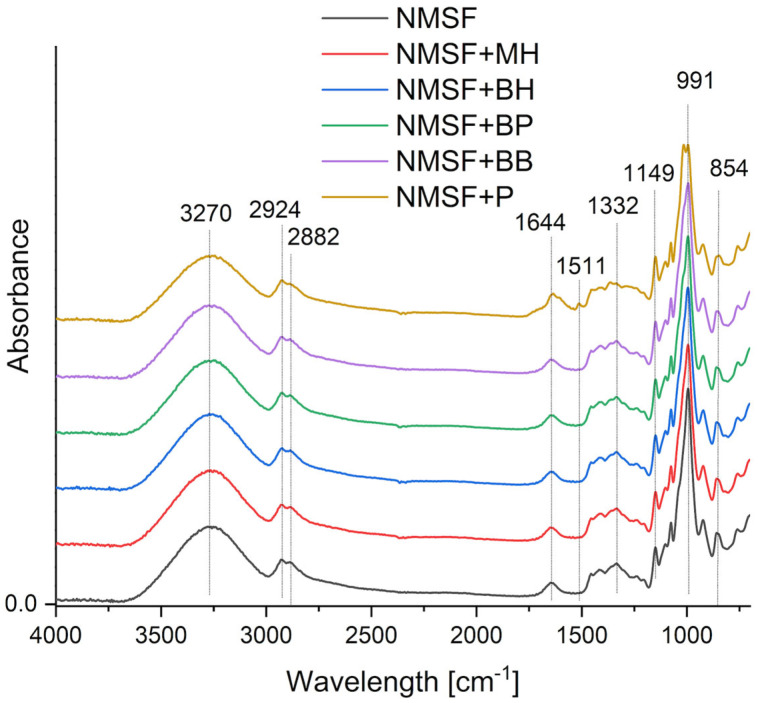
FTIR-ATR spectra of films based on native maize starch (NMSF) with or without extracts from honey-bee-derived products. Abbreviations of starch films: NMSF—native maize starch-based films, MH—multiflower honey, BH—buckwheat honey, BP—bee pollen, BB—bee bread, P—propolis.

FTIR-ATR spectroscopic analysis was performed for native and esterified maize starch-based films with or without extracts from honey-bee products. The FTIR-ATR spectra (Figure 1) of starch-based biopolymer films exhibited similar vibrational peaks corresponding to native maize starch. The FTIR-ATR spectrum of maize starch shows characteristic absorption bands, primarily in the regions of 3000–3500 cm^−1^, attributed to stretching vibrations of hydroxyl groups (–OH) present in starch and water adsorbed on its surface; 2800–3000 cm^−1^ (C–H stretching); and 1100–1300 cm^−1^ (C–O stretching and CH_2_ bending) [26]. The strong band at 2928 cm^−1^ corresponds to stretching vibrations of C–H bonds in the starch molecule [27]. The region between 1150 and 760 cm^−1^ is critical for identifying the starch backbone. This region contains bands caused by C–O–C vibrations, C–C vibrations, and other vibrational modes of glucose units and their linkages. The band near 1644 cm^−1^ may indicate the presence of absorbed water, which is common in starch samples.

The spectrum recorded for the NMSF+P sample (native maize starch film with propolis) differs from the control sample (NMSF—native maize starch-based film). In the spectrum of the propolis-containing sample, changes in band intensity within the 3000–3500 cm^−1^ range are observed, resulting from reduced water content. Additionally, a distinct intense absorption band at 1745 cm^−1^, assigned to ester bond stretching (C=O), and C–H deformation coupled with aromatic stretching at 1511 cm^−1^, attributed to flavonoids, are evident [28]. Similar results were reported in our previous study [11] on octenyl-succinylated potato starch-based films incorporated with honey-bee product extracts.

**Figure 2:**
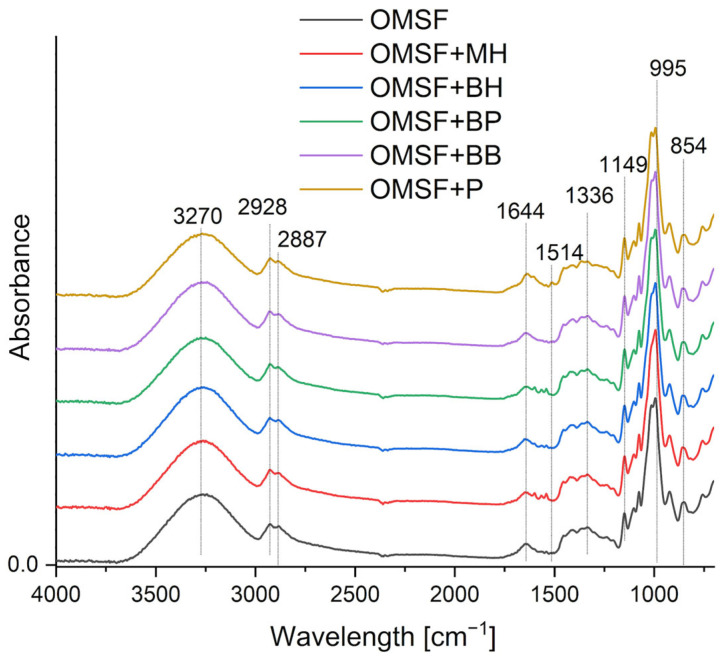
FTIR-ATR spectra of films based on OSA maize starch (OMSF) with or without extracts from honey-bee-derived products. Abbreviations of starch films: OMSF—octenyl-succinylated maize starch-based films, MH—multiflower honey, BH—buckwheat honey, BP—bee pollen, BB—bee bread, P—propolis.

A comparable effect is observed for the octenyl-succinylated maize starch film series with honey-bee product additives (Figure 2). All samples exhibit bands characteristic of octenyl-succinylated maize starch. The FTIR-ATR spectrum of octenyl-succinylated maize starch (OMSF) shows distinctive bands at 1723 cm^−1^ and 1563 cm^−1^, assigned to carbonyl (C=O) and carboxylate (RCOO^−^) groups, respectively, introduced via chemical modification [29]. As with the previous series, the OMSF+P sample (octenyl-succinylated maize starch film with propolis) displays characteristic propolis-derived bands. In the spectra of the OMSF+BP sample (octenyl-succinylated maize starch film with bee pollen), peaks in the 1610–1550 cm^−1^ range are visible. These bands can be attributed to amide I, an intense absorption band in proteins (β-sheet structure) associated with the protein fraction of bee pollen [30].

**Figure 3:**
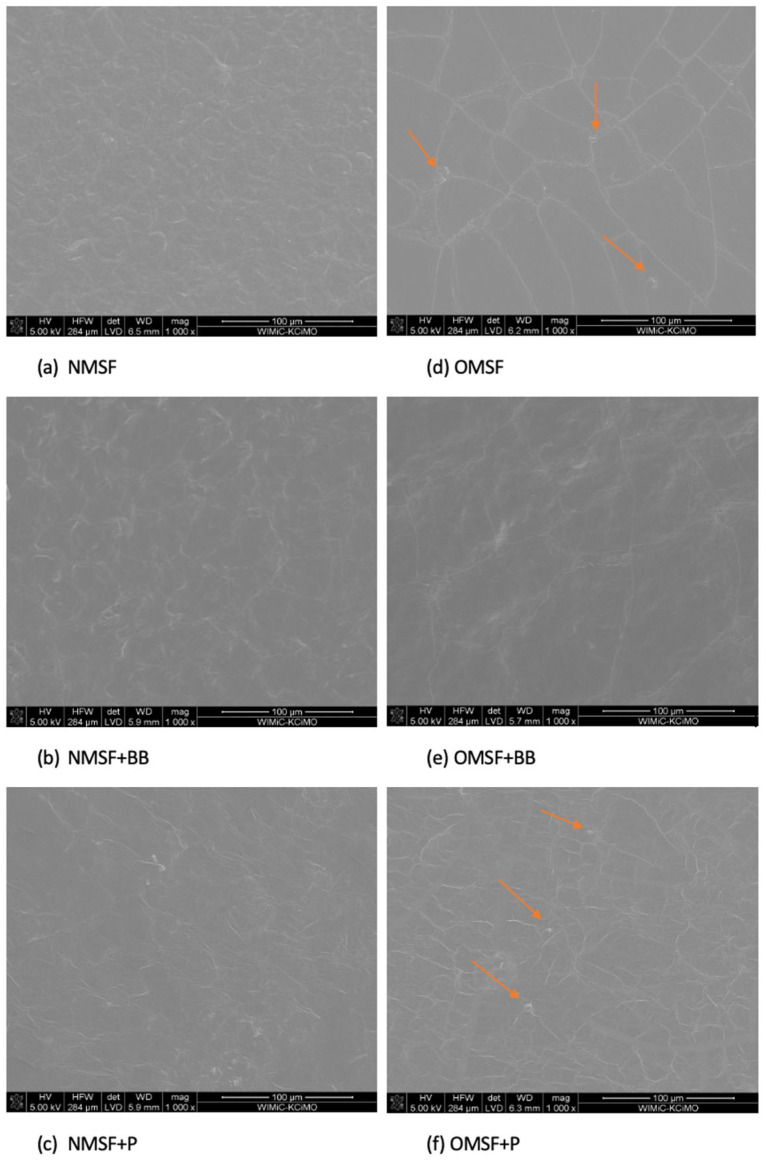
Surface SEM micrographs of films: (**a**) NMSF, (**b**) NMSF+BB, (**c**) NMSF+P, (**d**) OMSF, (**e**) OMSF+BB, (**f**) OMSF+P. Abbreviations of starch films: NMSF—native maize starch-based films, OMSF—octenyl-succinylated maize starch-based films, BB—bee bread, P—propolis.

Changes in band intensity (e.g., near 3270 cm^−1^) and slight positional shifts indicate structural modifications in the starch matrix due to additives. For honey-containing samples, increased absorbance in the -OH group region suggests enhanced hygroscopicity of the system, which may influence starch physicochemical properties, such as water-binding capacity [11]. Conversely, new bands in propolis- and pollen-containing spectra confirm the presence of non-starch compounds, implying limited compatibility of these additives with the polysaccharide matrix. It can be explained by the presence of hydrophobic compounds in HBE (such as flavonoids, phenolic acids, waxes, unsaturated and saturated fatty acids, sterols, terpens, volatile oils), which tend to phase-separate from the hydrophilic starch matrix [31]. This incompatibility results in surface roughness and discontinuities in the films, as observed in the SEM images (Figure 3). In summary, FTIR-ATR analysis allowed the identification of characteristic functional groups introduced by the additives and the tracking of their interactions with native and modified starch.

### 2.3. Scanning Electron Microscopy (SEM)

The structural morphology of films plays a crucial role in packaging characterization, as it directly influences key attributes like water vapor permeability and thermal behavior [32]. Scanning Electron Microscopy analysis revealed clear differences in the surface morphology of the starch-based films (Figure 3a–f). Film produced from native maize starch (NMS) (Figure 3a) exhibited folded, rough surface and structural irregularities were observed. These features indicated weaker internal cohesion and a potential limitation on the mechanical stability and barrier properties of NMS films. Similar microstructure of maize starch was reported by Thiré et al. [33]. The authors revealed the presence of both smooth and rough domains in maize starch film structure, but film morphology depended on the heating time used for gelatinization. In contrast, smooth surface with no pores and cracks and homogeneity in SEM cross-section was observed in films based on maize starch in a study reported by Garcia et al. [34], as well by Menchaca-Rivera et al. [35]. Also, other authors in AFM analysis report that maize starch films exhibited irregular surface with the presence of clusters of globular corpuscles and depressions [36].

Esterification process with using octenyl succinic anhydride changed the films morphology. OS starch films without HBE showed a more compact, uniform, and continuous surface structure than its counterpart prepared from native maize starch; however, some visible cracks and a few holes were observed on the OSF surface. The presence of octenyl succinate groups likely enhances film-forming ability and improves intermolecular interactions, resulting in fewer surface defects and a denser matrix. The modified films appeared to have a smoother and more uniform surface compared to the more folded structure observed in native maize starch-based film samples. As reported by Martins and Martins [32], starch crosslinked with citric acid allows for the production of uniform and cohesive films. During esterification, citric acid replaces hydroxyl groups in the starch molecules with its own carboxyl groups. This modification enhances the plasticizing efficiency of glycerol and contributes to improved surface cohesion and homogeneity of the resulting films.

However, after the incorporation of HBE (Figure 3), both type of films—those based on native and OS maize starch—exhibited a folded and rough surface. In OS starch films containing propolis, the surface appeared more cracked, with visible holes and disrupted structural integrity. In the context of the structural properties of starch films determined using SEM, some similarities were observed between the effects of propolis in this study and those for other bioactive additives rich in non-polar compounds, such as essential oils reported in the literature. The irregular and coarse surface, with non-uniform and heterogeneous structure, of the maize starch-based films with the addition of sunflower oil was also observed by Gao et al. [37]. The presence of obvious salient points through the SEM images was attributed to the location of oil droplets or incompletely broken starch granules. The particle breakage, rough structure, and formation of a discontinuous phase were also shown by Acevedo-Fani et al. [38] in alginate films containing essential oils. Also, Song et al. [39] reported that the addition of lemon essential oil to corn and wheat starch films at concentrations ranging from 0.5% to 2.0% inhibited particle breakage and led to the formation of a discontinuous phase of films. Chen et al. [23] observed that after incorporation of different phenolic compounds, the subsurface of maize starch films turned into granular protrusions, resulting in a decrease in the smoothness and flatness of the film surface. Wei, Li, and Li [40] reported that phenolic compounds with simple structure exhibit a weak capacity to combine starch by hydrogen bonds, whereas phenolic compounds with complex structure (such as more phenolic hydroxyl groups, larger molecular weight, and larger steric hindrance) can significantly interfere with the linear arrangement of amylose molecules, so as to increase the aggregation disorder of polysaccharides molecules.

### 2.4. Visual Characteristic and Optical Properties

The appearance and optical properties of films are integral to consumer acceptance of packaged food products [41]. As shown in Table 1, films based on native and OSA-modified maize starch exhibited comparable appearance and clarity. They display a slightly yellowish tint, which is typical for maize starch due to its higher lipid content compared to, for example, potato starch [17]. Films containing propolis extract appeared more aerated, with visible air bubbles on their surface. This is likely due to the octenyl succinylation of starch, which enhances its emulsifying and foaming properties—an expected outcome when using OS starch in film formation. The control samples (NMSF and OMSF) exhibited similar lightness values, and the addition of HBE did not significantly alter these parameters, except in the case of propolis. This additive caused a noticeable decrease in both the L* value and whiteness index, which aligns with findings from a previous study on potato starch-based films enriched with HBE [12]. A marked reduction in the a* coordinate (indicating a shift toward green tones) was observed after incorporating HBE into the film-forming solution, following this order: multifloral honey = buckwheat honey < bee pollen < bee bread < propolis. Propolis also caused a significant increase in the b* coordinate (indicating a shift toward yellow), leading to a substantial rise in the Yellowness Index compared to the control (from 4.3 to 53.5 in films with native maize starch, and from 4.9 to 52.4 in films with OS starch). The addition of bioactive compounds to the film polymer matrix can change the film color to varying degrees, which depends on the color of phenolic compounds [23]. The Total Color Difference between HBE-containing films and control samples (NMSF and OMSF, respectively) remained below 2 in most cases, suggesting that color differences were not visually noticeable. However, the inclusion of propolis led to a significant increase in ΔE*, which is consistent with literature reports on propolis incorporation into starch- or chitosan-based films [12,31].

### 2.5. Thickness Measurement

Film thickness is a very important parameter in determining film properties, particularly mechanical, thermal, and barrier characteristics. The films exhibited an average thickness ranging from 80 to 115 μm (Table 2). The lowest thickness was observed in both control films (NMSF and OMSF). Following the incorporation of honey-bee-derived extracts, a significant increase in thickness was observed only in the case of propolis for films based on native maize starch, and in the case of films containing buckwheat honey, bee bread and propolis for those based on OSA-modified starch. Generally, starch esterification slightly increased the film thickness, with the greatest effect observed in films with propolis addition. OS starch forms paste with higher viscosity than native starch [42], which may result in a thicker film layer. Another potential reason is the nature of molecular interactions. In OS starch, the presence of hydrophobic groups weakens hydrogen bonds between the hydroxyl groups of glucose residues. This disruption reduces the ability of starch molecules to pack tightly, leading to the formation of looser and less-ordered networks, which can further increase the film thickness. A significant increase in thickness observed in OS films with propolis addition may be related to interactions between the hydrophobic residues of starch OS and the lipids or polyphenols present in propolis. The hydrophobic groups of the HBE interact with the hydrophobic groups of OS starch, disrupting intermolecular interactions and spatial network formation, leading to thicker and less-ordered films. An increase in the thickness of maize starch films was also observed by Chen et al. [4] for films incorporated with naringin, protocatechuic acid, and tannic acid. In their study, the film thickness depended on the structure of the phenolic compounds—particularly the number of hydroxyl groups and their polarity—and consequently, their dispersibility in the film-forming solution. The increase in thickness was also greater with higher additive concentrations, ranging from 0.104 mm (for the control sample) to 0.141 mm (after the addition of naringin). It is worth mentioning that films prepared from maize starch with bee products were thinner than their potato starch counterparts, where the addition of, e.g., propolis and bee bread caused the thickness to exceed 130 µm [11]. This effect of maize starch may result from its lower water-binding capacity, which causes the films to retain less moisture during drying and, consequently, exhibit reduced thickness [43].

### 2.6. Moisture Content, Solubility in Water and Swelling Ratio

Table 2 summarizes the water-related behavior of starch films. Edible films that are meant to disintegrate quickly in the mouth require high solubility and strong swelling capacity, whereas materials designed as primary food packaging should be more hydrophobic. Even so, a limited dissolution just before consumption can still be desirable because it accelerates the release of active compounds into the food product [44]. The native- and OSA-modified starch films contained between 24.6 and 31.3% moisture, depending on the type of film. The addition of honey-bee extracts also had no obvious effect on water solubility of the films; solubility varied based on the type of starch and extract used. Native starch-based films generally became more soluble upon addition of HBE, except in the case of bee pollen. OS starch-based films exhibited increased solubility primarily upon incorporation of propolis, whereas the addition of buckwheat honey, bee pollen, and bee bread extract resulted in decreased solubility. The swelling ratio of starch-based films significantly increased in the presence of HBE, and this effect was further enhanced by OSA modification. Glycerol, used as a plasticizer, increases the film’s affinity for water by exposing additional hydrophilic hydroxyl groups that bind water through hydrogen bonding. Honey-bee-derived products act as co-plasticizers: their diverse polar and non-polar constituents interact differently with starch chains, and small polar molecules, in particular, draw water into the polymer matrix, creating more mobile, loosely packed regions [44,45]. Non-covalent forces between starch and phenolics—hydrogen bonding, hydrophobic contacts, electrostatic and ionic interactions—can yield either V-type amylose inclusion complexes or weaker, non-inclusive complexes [46]. Das, Dutta and Mahanta [47] reported that natural extracts (tea in their study) increased film solubility by leaching into the casting solution and forming water channels; our results suggest a similar, composition-dependent mechanism for honey-bee extracts. Moreover, these interactions also appear to depend on the type of phenolic compounds, the starch structure, and the experimental conditions. Overall, octenyl succinylation approximately doubled the swelling ratio, regardless of the HBE used—a well-known consequence of the higher viscosity and water-binding capacity of OS starch. We observed the same trend previously in potato starch-based films. This is attributed to hydrophobic interactions disrupting the starch network, which allows for greater water absorption and retention [48]. The hydrophobic OS groups further weaken hydrogen bonds between starch molecules, increasing free volume within the film. As a result, the film becomes more permeable to water, leading to higher swelling. A notable difference in the swelling ratio was observed between films made from maize OS starch and those prepared from potato OS starch. Potato OS starch films showed swelling ratios ranging from 9 to 13, depending on the type of bee-derived additive used [11]. This phenomenon may be attributed to the natural presence of phosphate groups in potato starch [49]. These hydrophilic groups enhance water binding, facilitating greater water uptake by the films. They may also partially compensate for the hydrophobic effect introduced by octenyl succinate residues. In maize starch, the levels of these groups are insignificant; therefore, the hydrophobic effect of OSA makes its films swell less.

### 2.7. Water Vapor Permeability (WVP)

Table 2 presents the WVP values for the film samples. The results suggest that there were no significant differences between the control films (based on native starch—NMSF, and esterified starch—OMSF) after the incorporation of HBE. An exception was the OMSF film with propolis, which exhibited the highest WVP value among all samples. Similarly, Norajit et al. [50] reported no significant differences in WVP values for alginate films containing different types of ginseng extracts. This can be explained by the fact that water vapor transmission generally occurs through the hydrophilic regions of the film and depends on the hydrophilic–hydrophobic balance of the film-forming solution’s components. Conversely, the inclusion of plasticizer (HBE acts, alongside glycerol, as a plasticizer) results in an increase in the free volume within the system. This promotes solvent mobility and enhances water diffusion through the polymer matrix, thereby reducing the barrier properties of the film. Moreover, SEM micrographs (Figure 3f) showed that the surface of OS starch films containing propolis appeared more cracked, with visible holes and disrupted structural integrity. This could be responsible for the higher WVP values and weak mechanical properties of this film compared to the control. These morphological defects of films indicate a loss of structural cohesion within the starch network. Such discontinuities inherently facilitate the passage of water vapor, which is consistent with the significantly elevated WVP observed for propolis-containing film. At the same time, the damaged structure helps to explain why the tensile strength in OMSF+P dropped so much (Table 3). When the film contains cracks and uneven areas, it cannot distribute stress properly, making it much more likely to break when stretched. This weakening effect may result not only from the physical disruption of the polymer network but also from the molecular interactions between propolis constituents and OSA-modified starch. Low-molecular-weight phenolic compounds present in propolis can infiltrate the polymer matrix and interfere with starch–starch hydrogen bonding. Their partial plasticizing action increases chain mobility, reducing intermolecular cohesion and further contributing to the loss of mechanical integrity [11,12].

An increase in WVP was also reported by Daudt et al. [51] after incorporating husk powder into a film-forming solution based on Brazilian pine seed flour. The authors suggested that the presence of pores in the film’s microstructure may have contributed to this effect. Similarly, Pineros-Hernandes [52] found that the addition of rosemary extract to cassava starch films increased WVP compared to films without the extract or with only a small amount. When film constituents consist of hydrophilic compounds such as protein, carbohydrates, and fibers, the interactions between them can led to increased intermolecular spacing and hydrogen bonding, which facilitates water diffusion through the film, thereby increasing its permeability. Furthermore, in addition to the hydrophilicity of the polymeric material, the thickness of the film also affects its water barrier properties. Thinner hydrophilic films are generally less permeable due to reduced affinity for water, whereas thicker films often show higher permeability [53]. This is consistent with our findings, where both NMSF and OMSF films with propolis—being the thickest among the samples (Table 2)—exhibited the highest WVP values.

An ideal film should exhibit low water vapor permeability when used as a packaging for fruits and vegetables, as these foods lose mass due to nutrient volatilization during storage, primarily as a result of water transpiration [53]. On the other hand, films with high WVP are desirable for packaging products that require moisture release from the package to prevent condensation and the growth of mold and bacteria. Examples of such products include dried or semi-dried fruits and vegetables, ripened cheeses, freshly baked bread, and other dehydrated products in which controlled evaporation is beneficial, such as fruit or vegetable chips. Therefore, the proper selection of packaging for a given product should take into account all of its key characteristics, including mechanical and optical properties, as well as the cost of the packaging material itself.

### 2.8. Mechanical Properties

As shown in Table 3, incorporation of HBE generally caused the decrease in tensile strength and increase in elongation at break; however, these changes were dependent on the type of starch and HBE.

According to relevant studies, mechanical properties strongly depend on the chemical structures of polyphenolic compounds. Phenolics with a small molecular size can lead to the proper formation of V-type inclusion complexes between starch and polyphenolic compounds and can thus enhance the strength of the polymer matrix, thereby improving the film’s resistance to external loads [54]. At the same time, the low number of phenolic hydroxyl groups and the small molecular size of phenolic compounds exhibit a limited ability to bind to polysaccharide side chains and promote further aggregation of these chains. As a result, its capacity to restrict the relative displacement of molecules is weakened, leading to a film with higher elongation at break. Due to the highly complex composition of extracts from bee products, it is difficult to clearly determine which phenolic compounds were responsible for the mechanical properties. Considering the total phenolic contents and phenolic profiles of multifloral honey, buckwheat honey, bee pollen, bee bread, and propolis extracts (presented in our previous study [12]), as well as the total phenolic content and antioxidant activity observed in maize starch films (Table 4), it was found that higher phenolic concentrations in HBE and in the films corresponded to lower tensile strength of the material. The TPC in propolis extract likely contributes to the more pronounced decrease in TS due to the plasticizing effect of low-molecular-weight phenolics and ethanol residues, which disrupt starch–starch hydrogen bonding to a greater extent than they reinforce intermolecular interactions. This is consistent with our data concerning the water-related properties of film with propolis (Table 2) and with data from our previous reports [30]. Propolis is composed mainly of small-molecule phenolic compounds, such as flavonoids, phenolic acids, and their esters. Our previous study revealed that the major phenolic acids identified in propolis were gallic, caffeic, *p*-coumaric, ferulic and protocatechuic acids, while among the flavonoids, pinobanksin, pinocembrin, chrysin, galangin, quercetin, kaempferol, and myricetin were detected. These molecules are relatively mobile within the polymer matrix and possess multiple polar functional groups capable of forming hydrogen bonds with starch chains. Their incorporation into the system likely disrupts the native starch–starch hydrogen bonding network. As a result, films with propolis exhibit a pronounced decrease in tensile strength, along with an increase in swelling power and WVP (Table 2), which is consistent with a plasticizing effect attributed to these small phenolic constituents. In contrast, bee pollen is rich in high-molecular-weight biomolecules, such as proteins, structural polysaccharides, and lipid-wax components [30]. These macromolecular constituents are less capable of diffusing into the starch network and tend to form dispersed aggregates within the matrix [31]. Their presence increases film heterogeneity and may create physical barriers that limit the penetration of water molecules, which explains their highest reduction in solubility and swelling ratio (Table 2). At the same time, the partial hydrophobicity of the lipid fractions further contributes to lowering the water affinity of the films.

The elongation at break of both NMSF and OMSF increased after incorporation of HBE (mainly bee pollen); however, the enhancement was least pronounced with the addition of the propolis extract. This extract was the richest in various bioactive compounds, which may interact with each other and reduce the overall effect. Moreover, phenolic compounds with a high number of hydroxyl groups and greater molecular weight may enhance the tensile strength of the films while simultaneously reducing EB, depending on their concentration. This effect may be attributed to the formation of a compact structure resulting from strong interactions and aggregation between polysaccharides and phenolic compounds. Such a structure increases strength of the material but limits its flexibility. Biratu, Woldemariam and Gonfa [55] reported that incorporation of honey and propolis into coffee pulp pectin-based edible films caused improvement in elongation at break when the concentrations of honey-bee products were below 20%. A further increase in honey or propolis content from 20% to 60% caused a decrease in %EB, as well as in the tensile strength of the films. Bee pollen increased the elongation at break more strongly than the other HBE. This effect can be attributed to the presence of proteins and simple sugars acting as natural softening agents, the hygroscopic nature of pollen components, which enhances bound-water content, and the lipid–wax fraction forming flexible microdomains within the starch matrix. Together, these features promote chain mobility and stress dissipation, resulting in a more elastic film structure.

As shown in Table 3, the use of octenyl-succinylated maize starch reduced the rigidity of the films by approximately two-fold. A similar phenomenon was observed in the case of films produced using esterified potato starch [11], although the tensile strength values of those films were significantly lower compared to the maize starch-based films. These findings are consistent with results reported by Kurdziel et al. [56] who demonstrated that starch esterification with octenyl succinic anhydride alters the starch structure, weakening intermolecular hydrogen bonds and causing disintegration within the starch granule. This looser starch structure may lead to decreased stiffness of the films, resulting in reduced tensile strength of the material [57].

To conclude, the amphiphilic character of octenyl-succinylated (OS) starch may interact differently with lipophilic or hydrophilic constituents of HBE. Propolis phenolics (many of which are moderately lipophilic) may associate more strongly with the hydrophobic succinyl groups, leading to a distinct plasticization pattern compared to the native starch matrix. Meanwhile, pollen constituents (proteins/lipids) may have a greater affinity to OS starch, resulting in reduced solubility and swelling.

### 2.9. Thermal Properties

The results of DSC measurements of the starch films are shown in Table 3. No endothermic peaks were observed below 100 °C (thermograms not shown), indicating complete gelatinization of the starch during film preparation at 95 °C. The endothermic peaks observed in the range of 216–276 °C correspond to the melting temperature (T_m_) of the crystalline regions within the starch films, immediately followed by thermal decomposition. The DSC parameters—onset temperature (T_o_), peak (T_p_, corresponding to the melting temperature, T_m_), end temperature (T_e_) of the melting transition, and melting enthalpy (ΔH)—decreased significantly for both starch types (native and modified) following the incorporation all honey-bee extracts. The extent of this decrease depended on the type of extract used, confirming the plasticizing effect of polyphenolic compounds on the starch films. The film prepared with native starch exhibited the highest characteristic melting temperatures. The incorporation of bee product extracts into the system significantly reduced the phase transition temperatures; however, no statistically significant differences were observed among the films enriched with different extracts. A notable decrease in the melting enthalpy of the native starch-based film was also observed upon the addition of bee product extracts. Nevertheless, no significant differences were found among the enriched samples, except for the film containing the propolis extract, which demonstrated a significantly lower enthalpy value compared to the others. This effect may be associated with the higher content of polyphenolic compounds in this sample (Table 4).

The replacement of native starch in the film formulation with octenyl succinic anhydride (OSA) starch led to a significant reduction in both the melting temperature and enthalpy values compared with the control film. Similarly, the incorporation of bee product extracts resulted in a further decrease in these thermal parameters, except for the sample enriched with bee pollen extract. This observation is consistent with the observed decrease in tensile strength and increase in elongation at break after the addition of HBE (Table 3). The observed decrease in both the melting temperature (Tₘ) and melting enthalpy (ΔHₘ) indicates a plasticizing effect of HBE, which disrupts the hydrogen bonding and crystalline structure of starch, resulting in less stable and more imperfect crystals that require less energy to melt. In general, the thermal characteristics of starch complexes are significantly altered by the addition of polyphenols, although they can vary depending on the complexation conditions (such as concentration, pH, and temperature) and the composition of honey-bee-derived products, which can differ in polyphenol content and type [58].

A decrease in thermal degradation temperatures following the complexation of starch with bioactive ingredients has been reported in several studies. For instance, such a reduction was observed in the case of wheat, potato, and pea starches complexed with tea polyphenols [59], as well as in maize starch films incorporated with various essential oils, as reported by Šuput et al. [60]. A possible explanation is that the hydroxyl groups abundant in tea polyphenols compete with the hydrogen-bonding sites between starch chains, thereby reducing the number of hydrogen bonds formed between adjacent glucose units. This disruption weakens the overall intermolecular bonding within the starch matrix, leading to decreased thermal stability of starch-bioactive compound blends. However, an opposite effect was reported by Zheng et al. [60] in the complexation of maize starch with caffeic acid. The authors observed an increase in the onset, peak, and conclusion temperatures of maize starch after complexation with caffeic acid, possibly due to reinforcement of the amylopectin structure and the restricted penetration of amylose. Nonetheless, a decrease in ΔH with increasing concentrations of caffeic acid may be attributed to water redistribution between caffeic acid and maize starch, as well as a reduced extent of the crystalline region after starch–caffeic-acid complexation. These differences are strongly influenced by alterations in the crystalline structure of starch resulting from the complexation process. In our previous study [11,12], we confirmed a reduction in the crystallinity of potato starch-based films after incorporating various honey-bee-derived product extracts. Polyphenols, due to their plasticizing effect, may interfere with starch chain associations through steric hindrance, thereby limiting crystal growth and recrystallization—findings that were also supported by our other results [12]. From a production standpoint, lower melting temperatures and reduced enthalpy of thermal transitions are advantageous, as they decrease the energy required to melt the material, for example, during sealing processes in sachet or bag manufacturing.

### 2.10. Total Phenolic Content and Antioxidant Activities Measured Using ABTS^•+^ (2,2′-Azino-bis(3-ethylbenzothiazoline-6-sulfonic acid)) and DPPH^•^ (2,2-diphenyl-1-picrylhydrazyl) Assays

Edible polymer films can serve as carriers for bioactive compounds. Honey-bee-derived products are promising agents in active packaging formulations due to their antioxidant, antimicrobial, antifungal, and antivirus properties [61]. While honey is widely used in culinary applications and pharmacy, other bee products such as bee bread, bee pollen, and propolis are still less popular and their applications remain limited. Thus, incorporating these natural products into food packaging materials (e.g., edible films) represents an interesting technological approach to utilizing them as antioxidant agents, with the aim of prolonging the shelf life of food products and enhancing health benefit for consumers. The antioxidant properties of the films are presented in Table 4. It was found that incorporating HBE generally increased the phenolic content and antioxidant activity of the films—particularly in the case of bee pollen, bee bread, and most notably, propolis extracts. The total phenolic content of the films with propolis extract was almost twice as high as reported by Salimi et al. [62] for films made from apple pomace pectin and grass pea protein incorporated with 12% propolis extract (14.59 mgGAE/g film). The difference may be attributed to variations in the extract preparation procedure, as well as to the concentration of propolis in the film-forming solution. Films containing multifloral and buckwheat honeys exhibited the lowest TPC and antioxidant activity values, although buckwheat honey appeared to offer slightly better antioxidant properties than multifloral honey. Similar results were observed in the study on potato starch-based films incorporated with honey-bee-derived extracts [12]. Velásquez et al. [63] did not observe any significant differences in TPC among k-carrageenan films incorporated with equal amounts of ulmo or quillay honeys. However, the same authors reported that films with bee pollen extract had a four-fold higher TPC compared to those with honey, likely due to the specific composition of phenolic acids and flavonoids.

The films prepared on octenyl-succinylated maize starch exhibited higher values of TPC and antioxidant activity than films based on native maize starch, particularly in the case of bee pollen, bee bread, and propolis. This is consistent with our previous study on potato starch-based films [12]. The results demonstrated that hydrogen bonding and hydrophobic interactions between HBE and octenyl-succinylated starch contributed to the improved antioxidant activity and total phenolic content of the films. It can be stated that starch modification through the introduction of hydrophobic moieties transforms the granule into an amphiphilic structure, which can more effectively interact with the bioactive compounds in HBE. Improved TPC and antioxidant activity were also observed by Bahraminejad et al. [64] in OSA-modified alginate bead samples incorporated with bergamot essential oil. The authors reported that modified alginate was more capable of encapsulating bergamot essential oil, increasing its concentration in the inner fraction of the beads, while simultaneously reducing the amount retained on the surface. Similarly, in the case of maize starch films with HBE, the introduction of OS groups likely facilitated the formation of inclusion complexes involving hydrophobic interactions, as well as non-inclusion complexes stabilized by water-binding forces [58].

### 2.11. Antimicrobial Properties

The films used in the experiment showed a varied zone of inhibition of microbial growth, ranging from 0 to 25 mm, depending on the type of HBE used (Table 5). The lack of antimicrobial properties against all tested strains of Gram-negative bacteria and most strains of Gram-positive bacteria was demonstrated by the control films produced using native (NMSF) or octenyl-succinylated maize starch without any extracts (OMSF), as well as films enriched with multiflower honey or buckwheat honey. A small degree of growth inhibition (zone with a diameter of 7.3 mm) was noted only in the case of OMSF+ BH in relation to *S. equorum*.

Films with the addition of bee bread extract showed quite a significant inhibition against four (NMSF) or five (OMSF) strains of bacteria; inhibition zones were ranging from 11 to 25 mm and from 8.3 to 25 mm, respectively. Both types of films with the addition of bee pollen extract inhibited the growth of four bacterial strains to a small extent (zones in the range of 4.5–12 mm), and showed the strongest effect against *S. xylosus*. Contrary to our studies, Pełka et al. [65] observed a slightly lower activity of bee pollen and bee bread against Gram-negative bacteria compared to Gram-positive staphylococci. The cited authors showed a higher antibacterial efficiency of bee bread compared to bee pollen against staphylococci, which is consistent with our results.

The most comprehensive and strongest bacteriostatic effect on both Gram-positive and Gram-negative bacteria (for seven out of nine tested strains of staphylococci, *Salmonella* and *E. coli*) was observed for both types of films with the addition of propolis, recording growth inhibition zones in the range of 9.5–25 mm. Propolis had the strongest bacteriostatic effect against one of the tested strains of *S. aureus* and *Salmonella*, which is excellent information from the therapeutic point of view. The bacteriostatic activity of propolis against *S. aureus* has been reported by many researchers, e.g., [66,67]. It should be noted that in the cited studies, propolis acted only above a certain concentration; at low concentrations, the effect was not observed. Similar results were obtained in our previous work, in which propolis was embedded in octenyl-succinylated potato starch-based films [12].

Among the Gram-positive staphylococci tested, *S. chromogenes* did not show sensibility to any of the products tested; 6 out of 10 tested products with added bee products were effective against *S. xylosus*. In the case of Gram-negative bacteria, a strong bacteriostatic effect of the film with the addition of propolis was found on the *Salmonella* strain; on *E. coli* films with the addition of propolis and bee bread, extracts had a bacteriostatic effect. Grecka et al. [68] tested ethanolic propolis solutions and stated that there was no antimicrobial activity against *E. coli* up to a concentration of 4096 µg/cm^3^. Staphylococci (*S. aureus* and *S. epidermidis*) showed much higher and even over 100-fold sensitivity (from 32 to 256 µg/cm^3^).

Films with bee bread and propolis extracts had a strong bacteriostatic effect on the Gram-positive bacterium *Bacillus subtilis*. Sani et al. [69] also observed strong activity of propolis in ethanolic solution against *Bacillus subtilis*, determining an MIC of 156.25 µg/cm^3^. Interesting in this context are the results obtained by Pełka et al. [70], who examined bee bread and bee pollen in terms of their potential to inhibit selected human pathogenic bacteria. For this purpose, they isolated 81 bacterial strains from these two bee products, of which as many as 42% showed antagonistic interactions with at least one reference strain of pathogenic bacteria (including *Staphylococcus aureus*, *S. epidermidis*, and *E. coli*). All strains producing antimicrobial agents belonged to the genus *Bacillus* and among them, *Bacillus subtilis* used in our studies.

### 2.12. Apple Storage

**Figure 4:**
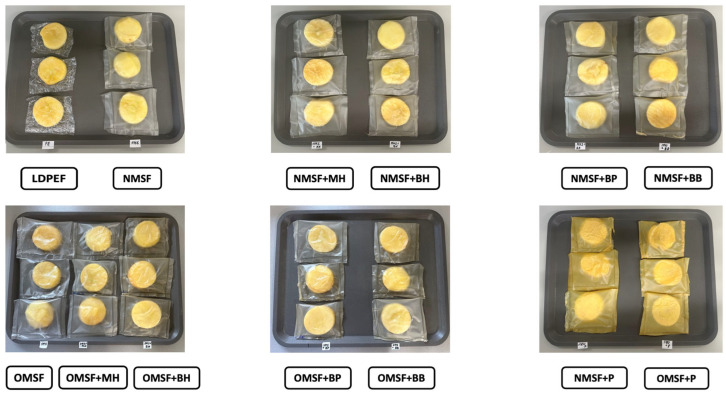
Fresh apple slices packed in films based on native and octenyl-succinylated maize starch-based films with HBE. Abbreviations: NMSF—native maize starch-based films, OMSF—ocetynyl-succinylated maize starch-based films, MH—multiflower honey, BH—buckwheat honey, BP—bee pollen, BB—bee bread, P—propolis, LDPEF—low-density polyethylene film.

**Figure 5:**
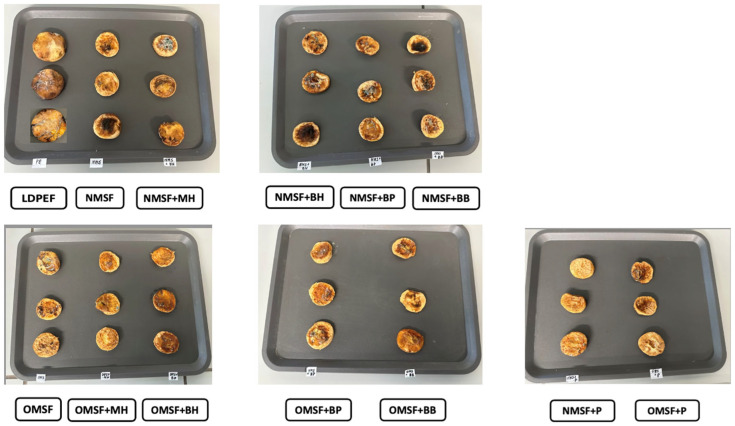
Apple after 7 days of storage in films based on native and octenyl-succinylated maize starch-based films with HBE. Abbreviations: NMSF—native maize starch-based films, OMSF—ocetynyl-succinylated maize starch-based films, MH—multiflower honey, BH—buckwheat honey, BP—bee pollen, BB—bee bread, P—propolis, LDPEF—low-density polyethylene film.

Typically, parameters such as appearance, weight loss, color changes, and water activity are evaluated to assess the freshness and overall quality of fresh-cut fruits during storage. Commonly, fresh fruits are stored in synthetic films; however, they are prone to rapid mold growth, which can be attributed to the low permeability of these materials to water vapor during the respiration process. As shown in Figure 4 and Figure 5, apple slices were individually packed and sealed in starch-based films (in triplicate). To compare the effects of the edible films on fruit quality, an additional control group was packed in commonly used LDPE films.

After 7 days of storage at ambient temperature, all apple samples exhibited a noticeable decline in quality compared to fresh apples. The lightness (L*) value decreased substantially from 79.9 (for fresh apples) to 37.4 for fruits stored in NMSF films containing buckwheat honey (Table 6). In contrast, samples stored in NMSF with propolis and in LDPE films showed significantly higher L* values after storage (L* = 47.8 and L* = 52.2, respectively). Similar findings were reported by Pastor et al. (2011) in the case of grapes coated with hydroxypropyl methylcellulose edible coatings containing propolis, but only with its highest concentration [71]. The samples after storage time were the lightest, probably due to the greatest film opacity [71]. Enzymatic browning caused by polyphenol oxidase, as well as non-enzymatic browning, can make fresh-cut fruits darker and reduce their organoleptic quality and commercial value [23,71]. The browning index (BI) increased markedly compared to both fresh and synthetically packed apples. Notably, higher BI values were observed for samples stored in films enriched with bee bread and propolis. extracts, which may be attributed to the migration of natural colorants (e.g., phenol compounds) from the film matrix to the fruit surface. This observation is consistent with findings reported in other studies [5,72]. Water activity (aw) decreased along with the observed increase in weight loss. Fresh-cut apples initially exhibited a water activity of 0.854. After storage, a significant reduction in aw was observed across all samples, with the largest decrease recorded for apples packed in OS starch films containing propolis. After 7 days, the weight loss of apple samples ranged from 55.48% to 63.04%, with slightly higher values for fruits stored in octenyl-succinylated starch films, likely due to the presence of air bubbles observed in their structure.

Apples packed in synthetic LDPE films exhibited the least overall quality deterioration (BI = 76, weight loss = 2.37%, aw = 0.850, comparable to fresh samples). However, these samples showed the most extensive mold growth and developed a mucilaginous consistency. In contrast, apples stored in edible starch films also presented mold spots, but their texture was gummier and more dehydrated. The number and size of mold colonies assessed visually decreased after incorporating honey-bee extract, and in the case of OMSF films enriched with propolis, no mold growth was observed on the apple surface. This observation may be attributed to the remarkable antimicrobial and antioxidant activity of films containing propolis (Table 4 and Table 5). Pastor et al. (2011) reported no significant effect of edible coatings on maintaining the storage quality of grapes [71]. However, despite the lack of such effects on grape quality, the incorporation of propolis enhanced the nutritional attributes of the coated fruit, suggesting its potential for developing healthier products [71].

Storage tests of apple slices showed that the films, due to their hydrophilic nature associated with the hydrophilic matrix of starch, are not suitable for storing high-moisture foods. Even the addition of propolis, which contained the highest number of phenolic compounds and exhibited the greatest microbiological potential, did not produce the expected effects. Such a significant loss of apple mass and drastic change in color, even in the absence of microbiological spoilage, would not be acceptable to consumers. However, the results suggest that selecting a more suitable product for storage—e.g., one with lower water content or more hydrophobic in nature, such as fresh or smoked meat, fish, yellow-orange cheeses, or oils—may prove much more effective than apples. Films with high WVP can still be useful in applications where moisture exchange is beneficial, such as packaging of produce that requires respiration (e.g., certain fruits and vegetables), where controlled water vapor transmission can help reduce condensation and extend shelf life. Furthermore, starch films with the addition of HBE could serve as a natural barrier against photo-oxidation of food, as well as one of the layers in a multilayer packaging system providing better moisture barrier properties. The multilayer packaging systems are also a novelty on the market and requires thorough investigation.

## 3. Materials and Methods

### 3.1. Materials

N-octenyl succinic acid anhydride (n-OSA) was obtained from Sigma-Aldrich (St. Louis, MO, USA). Glycerol was supplied from Chempur (Piekary Śląskie, Poland). Native maize starch (NMS), with a dry mass (d.m.) of 88.76%, was purchased from Cargill Poland (Warsaw, Poland). Bacterial strains from the ATCC collection (American Type Culture Collection) were acquired from Biomaxima (Lublin, Poland), as follows: St1—Staphylococcus sciuri (ATCC 29061), St2—Staphylococcus xylosus (ATCC 29967), St3—Staphylococcus succinus (ATCC 7003), St4—Staphylococcus vitulinus (ATCC 51145), St5—Staphylococcus equorum (ATCC 43958), St6—Staphylococcus epidermidis (ATCC 12228), St7—Staphylococcus chromogenes (ATCC 43764), St8—Staphylococcus aureus (ATCC 25923), St9—Staphylococcus aureus (ATCC 29213), Sal—Salmonella enterica subsp. Enterica ser. Enteritidis (ATCC 13076), Ec—Escherichia coli (ATCC 25922), Bs—Bacillus subtilis (ATCC 6633). Four bee-derived products were collected from local apiaries in the Malopolska region (Poland), including multifloral honey (MH), buckwheat honey (BH), bee pollen (BP), and bee bread (BB). A concentrated ethanolic extract of commercial propolis (P) was sourced from the Bio-Pharmaceutical Laboratory (Krakow, Poland). Rubin apples were obtained from a local shop (Krakow, Poland).

### 3.2. Methods

#### 3.2.1. Preparation of Octenyl-Succinylated Starches and Quantitative Determination of the Effectiveness of Esterification Process

Octenyl-succinylated maize starch (88.29% d.m.) was prepared following the method described by Hui et al. [73]. The esterification was performed using a 35% (*w*/*w*) starch–water suspension. For esterification, 7% (*v*/*v*, based on d.m. of starch) of OSA was used. The OSA solution, diluted five-fold with absolute ethanol, was gradually added to the starch suspension over 1 h. The esterification reaction was conducted for an additional 4 h, and the entire procedure was repeated three times. The resulting modified OS starch was subsequently dried, milled, and sieved through a 125 μm mesh sieve (AS200, Retsch, Germany). The obtained material was then averaged.

The degree of substitution (DS) of OS starch was determined by titration according to the classical method of Hui et al. [73].

#### 3.2.2. The Extraction Procedure of Phenolic Compounds

The honey-bee extracts were prepared according to the method outlined in our previous paper [11]. Each honey-bee product (15 g) was extracted by shaking with 50 mL of 80% ethanol (*v*/*v*) at 50 °C for 20 h. The samples were then sonicated at 40 kHz for 30 min at 30 °C. The extracts from three repetitions were combined, filtered, and stored at 6 °C until further analysis.

#### 3.2.3. Films Preparation

Starch films were prepared following the methodology described in our previous study [16]. A 2% (*w*/*w*) starch dispersion (based on dry matter content) was heated to 95 °C for 30 min with continuous stirring at 300 rpm. Afterward, glycerol was gradually added as a plasticizer at a concentration of 1% (*w*/*w*) of the total mixture, and the dispersion was stirred for an additional 10 min. A 50 g portion of the cooled paste was then poured into a plastic Petri dish (14 cm outer diameter) and dried under controlled conditions (60 °C and 50% relative humidity (RH)). Films containing ethanolic extracts of honey-bee products were prepared using the same procedure, except that 1 g of the respective HBE was incorporated into the 100 g of the cooled film-forming solution. The mixture was thoroughly stirred for 15 min before being poured into the plates for drying. To ensure even distribution of the propolis extract within the polymer matrix, it was introduced into the film-forming solution as a 20% (*v*/*v*) ethanol solution. The starch-to-glycerol-to-propolis extract ratio was consistently maintained at 2:1:1 (*w*/*w*/*w*), corresponding to 2 g of starch (dry matter), 1 g of glycerol, and 1 g of HBE per 100 g of mixture. The same proportions were used in formulations with other HBE. Each film formulation was prepared in twenty repetitions. Finally, the samples were stored in a desiccator at 20 ± 2 °C and 53 ± 2% RH until further analysis.

#### 3.2.4. Characterization of Starch Films

1. Fourier Transform Infrared Spectroscopy with Attenuated Total Reflectance (FTIR-ATR)

Fourier Transform Infrared Spectroscopy was used to analyze the structural interactions between maize starch and the HBE compounds within the films according to the procedure described by Królikowska et al. [74].

The FTIR-ATR spectra of native and octenyl-succinylated maize starch-based films were collected over the wavenumber range of 4000–700 cm^−1^ with a resolution of 4 cm^−1^. Measurements were performed using a Mattson 3000 FTIR spectrophotometer (Madison, Wisconsin, USA) equipped with 30SPEC 30-degree reflectance adapter and a MIRacle ATR accessory from PIKE Technologies Inc. (Madison, WI, USA). Film samples were analyzed directly in contact with the ATR crystal without further preparation.

2. Scanning Electron Microscopy (SEM)

SEM images of films were taken with a Nova Nano Sem 200 (Fei Europe Company, Zaventem, Belgium) electron microscope. The analysis was only performed for samples that exhibited the most distinct differences in their physicochemical properties, including the control films (NMSF and OMSF) and the films containing bee bread and propolis. Film pieces were mounted on aluminum stubs using double-sided tape and then coated with a thin film of gold. The surface morphology of the films was observed directly at an accelerating voltage of 10 kV and a magnification of 1000×.

3. Visual characteristic and optical properties

Films were assessed visually, while their optical properties were evaluated in the CIE Lab color system using transmission coordinates, where L* denotes lightness, a* reflects the red–green axis, and b* corresponds to the yellow–blue axis. Measurements were performed using an spectrophotometer X-Rite Color i5 (X-Rite, Grand Rapids, MI, USA) with a 10º/D65 illuminant/observer setting and a d/8° geometry configuration [20] Data were collected from five different locations on film surface, and the mean values were calculated. The total color difference (ΔE), along with whiteness index (WI) and yellowness (YI) index were determined using the following equations:

ΔE* = [ΔL*^2^ + Δa*^2^ + Δb*^2^]^1/2^ (1)

WI = 100 − ([100 − L]^2^ + a^2^ +b^2^)^0.5^ (2)

YI = 142.86b/L(3)

where

ΔL* = L*_samlple_ − L*_reference_

Δa* = a*_samlple_ − a*_reference_

Δb* = b*_samlple_ − b*_reference_

L*, a*, b*—mean values of the respective CIELab coordinates for the sample,

L*_reference_, a*_reference_, b*_reference_—corresponding values of the reference (control) film.

4. Thickness measurement

Film thickness was measured using a digital thickness gauge (Insize Co., Ltd., Suzhou, China). Measurements were taken with an accuracy of 0.001 mm at ten random locations on each film sample, and the mean film thickness was calculated.

5. Moisture content, solubility in water and swelling ratio

Film samples measuring 20 × 20 mm were weighed (m_0_) and dried at 100 °C for 24 h, and then weighed with an accuracy of 0.0001 g to determine the initial dry mass (dm_0_). Next, the films were placed in conical flasks with water and shaken for 24 h at 25 °C. After soaking, the films were filtered, rinsed with distilled water, and weighed to obtain the mass of the swollen film (m_1_). Subsequently, the films were dried again at 100 °C for 24 h to determine the final dry mass (dm_24_). The water solubility and swelling ratio of the films were calculated using the following formulas [20]:

Moisture content (%) = (m_0_ − dm_0_)/dm_0_ × 100 (4)

Solubility (%) = (dm_0_ − dm _24_)/dm_0_ × 100 (5)

Swelling ratio (−) = (m_1_ − dm_24_)/dm_24_ (6)

6. Water vapor permeability (WVP)

Water vapor permeability was measured using the ASTM E 96-95 [75] standard (wet cup method). Film samples were sealed over cups containing distilled water, which acted as a moisture source. The cups were placed in a controlled environmental cabinet containing saturated magnesium nitrate (Mg(NO_3_)_2_) to maintain a relative humidity of ~53%. The partial pressure difference (PA_1_ − PA_2_) across the film was calculated from the known vapor pressure of water at 32 °C and the relative humidity inside and outside the cup. Water vapor transmission rate (WVTR) and WVP were calculated as follows:

WVTR = slope/film area(7)

WVP = WVTR × L/(PA_1_ − PA_2_) (8)

where PA_1_—vapor partial pressure at film outer surface in a cabinet, PA_2_—vapor partial pressure at film inner surface in a cup, and L—average film thickness.

7. Mechanical properties

The tensile strength (TS) and percent elongation at break (%EB) of the films were measured by using an EZ-SX Texture Analyzer (Shimadzu, Kyoto, Japan). Prior to analysis, the film was cut into rectangular strips (25 × 80 mm) and fixed by grids. The initial grip separation was set at 50 mm and the stretching speed was set at 50 mm/min [75]. Measurements were performed at 23 ± 2 °C on samples equilibrated at 53% RH.

8. Thermal properties

The thermodynamic properties (melting transitions) of the films were analyzed using a differential scanning calorimeter 204F1 (Netzsch, Selb, Germany), following the method described by Jamróz, Juszczak, & Kucharek [76]. Approximately 1 mg of each film sample was sealed in an aluminum pan and analyzed under a nitrogen atmosphere at a flow rate of 20 mL/min. The sample was heated from 30 °C to 300 °C at a rate of 10 °C per min. An empty pan was used as the reference. The onset (T_o_), peak (T_p_), end (T_e_) temperatures, and enthalpy change (ΔH) were recorded from the endothermic peak corresponding to the melting starch-based films. All films were previously conditioned to minimize thermal history effects.

9. Total phenolic content and antioxidant activities measured using ABTS and DPPH assays.

The total phenolic content (TPC) and antioxidant activity (AA) of the starch films were evaluated according to the methods outlined in [12]. Before analysis, 200 mg of the film (cut into pieces) was extracted by shaking with 5 mL of 80% (*v*/*v*) ethanol at 25 °C for 24 h.

To determine TPC, 0.5 mL of the ethanol extract was reacted with the Folin–Ciocalteu reagent. The mixture was allowed to incubate for 2 h, after which the absorbance was measured at 760 nm against a blank (80% *v*/*v* ethanol) using a UV/Vis spectrophotometer V-630 (Jasco, Tokyo, Japan). The results were expressed as mg of gallic acid equivalents (GAE) per g of d.m. of the starch films.

The ABTS cation radical scavenging activity of the ethanolic extracts of films was evaluated according to the procedure described by [12] The ABTS^●+^ was produced by reacting a 2 mM phosphate-buffered saline (PBS) solution of ABTS with potassium persulfate. For the assay, 100 μL of appropriately diluted extract was mixed with 3 mL of the ABTS^●+^ solution. After 15 min of incubation, absorbance was measured at 734 nm. The antioxidant activity (AA) was expressed as mg of trolox equivalents (TE) per g of d.m. of the starch film.

The DPPH radical scavenging activity was determined as follows: 3.7 mL of a 0.05 mM DPPH^●^ solution in methanol was mixed with 0.3 mL of the ethanolic extract of the film sample. After a 60 min of incubation, absorbance was measured at 515 nm. The antioxidant activity was expressed as mg of TE per g of d.m. of the starch film.

10. Antimicrobial properties

The antimicrobial properties of starch films enriched with HBE were evaluated using the disc diffusion method described in [67]. The test included nine bacterial strains: Gram-positive species, such as Staphylococcus sp. and Bacillus subtilis, as well as Gram-negative strains, including Escherichia coli and Salmonella enterica subsp. Enterica ser. Enteritidis. These microorganisms were selected due to their relevance as common foodborne pathogens and contaminants.

For the assay, bacterial suspensions were adjusted to match a 0.5 McFarland standard, using a DEN-1 densitometer (Biosan SIA, Riga, Latvia). Sterile plastic Petri dishes (90 mm) were prepared by pouring in 16 mL of Mueller–Hinton agar (Biomaxima Poland, Lublin, Poland). After the agar had solidified, 200 µL of each bacterial suspension was spread evenly over the surface. Starch film samples were cut into discs, 7 mm in diameter, and placed on the inoculated agar. Each dish contained four film discs and was incubated at 37 °C for 48 h. Following incubation, the diameters of the inhibition zones around the discs were measured to assess bacterial growth suppression.

#### 3.2.5. Apple Storage

For evaluation, apples were washed, peeled, and cut into slices of uniform size, approximately 5 cm in diameter and 1.5 cm in height. The apple slices were wrapped in pieces of the prepared films and then heat-sealed into sachets using a manual sealer (Hangzhou A Link Star Technology Co., Ltd., Hangzhou, China). The samples were stored for 7 days under ambient conditions (25 ± 2 °C, atmospheric air).

1. Visual appearance—the visual appearance of fresh and stored apple slices was assessed.
2. Color measurements—the L*, a*, and b* parameters were determined as described in Section 3.2.4. Data were collected from six different locations on the apple surface, and mean values were calculated. The browning index (BI) was calculated using the following equations [5]:

BI* = 100 (x − 0.31)/0.172

where

x = a* + 1.75L*/5.645L* + a* − 3.012b*

3. Water activity—the water activity (aw) of fresh and stored apple slices was measured using Swift Lab water activity meter (Swift Lab, Lachen, Switzerland).
4. Weight loss—weight loss during storage was determined by comparing the initial and final weights of the samples.

### 3.3. Statistical Analysis

The data underwent one-way analysis of variance (ANOVA), followed by the calculation of the least significant difference (LSD) using Fisher’s test at a significance level of 0.05. The mean values were derived from at least three measurements. Statistical analyses were conducted using the Statistica 10.0 software package (StatSoft, Kraków, Poland).

## 4. Conclusions

Honey-bee-derived products, particularly bee pollen, bee bread, and propolis, appear to be promising bioactive additives for the preparation of edible films. Their type and concentrations in the film-forming solution should be carefully tailored to the intended application. In this study, fresh-cut apples were used as a model food product. The incorporation of OS maize starch into starch–HBE formulations enhanced the functional and bioactive properties of the films, especially when combined with bee-derived products. Propolis-enriched films exhibited the highest phenolic content, antioxidant activity, and antimicrobial effectiveness, confirming their potential as active packaging materials. Although no significant effects on the quality of fresh-cut apple were observed, the incorporation of HBE, particularly propolis, contributed to extending the shelf life of fruit, highlighting its potential not as a standalone alternative to synthetic films, but rather as a component of a multilayer system with more hydrophobic characteristics. Further research is needed on starch-based films with varying concentrations of HBE to gain a better understanding of the interaction mechanisms between starch and HBE, as well as their influence on the functional properties of films. Future studies should also address the storage quality of different types of food products, such as fish, meat, or oils, where the yellow coloration of the films is less important for consumers than prolonged freshness.

## Figures and Tables

**Table 1:** Appearance and optical properties of native and octenyl-succinylated maize starch-based films with HBE.

Film Samples ^2^	Film Appearance	L* ^1^	a*	b*	ΔE*	Whiteness Index (WI)	Yellowness Index (YI)
NMSF (control)	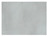	86.6 ± 1.3 ^bc^	−0.39 ± 0.01 ^a^	2.63 ± 0.11 ^c^	–	86.3	4.3
NMSF+MH	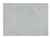	86.5 ± 1.0 ^bc^	−0.45 ± 0.05 ^a^	2.47 ± 0.06 ^c^	0.85 ± 0.28 ^c^	86.3	4.1
NMSF+BH	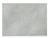	87.0 ± 0.6 ^ab^	−0.43 ± 0.04 ^a^	2.74 ± 0.03 ^c^	0.54 ± 0.45 ^c^	86.7	4.5
NMSF+BP	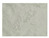	87.8 ± 0.2 ^a^	−0.74 ± 0.02 ^b^	3.93 ± 0.07 ^b^	1.93 ± 0.22 ^b^	87.2	6.4
NMSF+BB	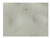	86.8 ± 0.2 ^b^	−0.61 ± 0.02 ^b^	3.60 ± 0.10 ^b^	1.02 ± 0.07 ^c^	86.3	5.9
NMSF+P	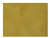	85.8±0.5 ^c^	−4.35 ± 0.25 ^c^	32.14 ± 1.14 ^a^	29.79 ± 1.13 ^a^	64.6	53.5
OMSF (control)	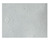	86.1 ± 0.2 ^D^	−0.28 ± 0.05 ^A^	2.96 ± 0.14 ^B^	–	85.8	4.9
OMSF+MH	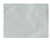	86.5 ± 0.3 ^BC^	−0.31 ± 0.04 ^A^	2.71 ± 0.27 ^B^	0.51 ± 0.33 **^B^**	86.2	4.5
OMSF+BH	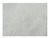	86.4 ± 0.1 ^CD^	−0.33 ± 0.03 ^A^	3.11 ± 0.25 ^B^	0.38 ± 0.18 ^B^	86.0	5.1
OMSF+BP	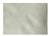	86.8 ± 0.3 ^AB^	−0.61 ± 0.03 ^B^	3.02 ± 0.16 ^B^	0.79 ± 0.23 ^B^	86.4	4.9
OMSF+BB	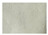	87.0 ± 0.2 ^A^	−0.59 ± 0.02 ^B^	3.25 ± 0.05 ^B^	1.00 ± 0.16 ^B^	86.6	5.3
OMSF+P	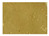	83.1 ± 0.5 ^E^	−2.48 ± 1.49 ^C^	30.46 ± 1.49 ^A^	27.76 ± 1.51 ^A^	65.1	52.4
LSD_0.05_	–	0.69	0.12	0.65	0.69	–	–

^1^ Within columns, values subscribed by the same small or capital letters do not differ significantly at *p* < 0.05. Data are means of six determinations. LSD_0.05_—least significant difference at significance level 0.05 calculated within column for group of all samples. ^2^ Abbreviations of starch films: NMSF—native maize starch-based films, OMSF—octenyl-succinylated maize starch-based films, MH—multiflower honey, BH—buckwheat honey, BP—bee pollen, BB—bee bread, P—propolis.

**Table 2:** The results of thickness and water barrier properties of native and esterified (OSA) maize starch-based films with or without honey-bee extract.

Film Samples ^2^	Thickness ^1^ [μm]	Moisture Content [%]	Solubility in Water [%]	Swelling Ratio [-]	WVP [g/m·s·Pa] × 10^−10^
NMSF (control)	80 ± 9 ^b^	29.1 ± 1.4 ^a^	20.6 ± 1.5 ^c^	2.61 ± 0.11 ^e^	1.52 ± 0.08 ^ab^
NMSF+MH	84 ± 5 ^b^	26.6 ± 1.3 ^b^	37.2 ± 2.0 ^a^	3.77 ± 0.26 ^c^	1.41 ± 0.08 ^b^
NMSF+BH	86 ± 5 ^b^	29.2 ± 2.0 ^a^	23.5 ± 0.6 ^b^	3.38 ± 0.21 ^d^	1.54 ± 0.10 ^ab^
NMSF+BP	84 ± 7 ^b^	28.6 ± 1.1 ^a^	10.9 ± 0.6 ^d^	2.30 ± 0.05 ^e^	1.40 ± 0.08 ^b^
NMSF+BB	84 ± 5 ^b^	29.8 ± 0.7 ^a^	23.7 ± 1.0 ^b^	4.57 ± 0.23 ^b^	1.40 ± 0.08 ^b^
NMSF+P	96 ± 11 ^a^	24.4 ± 0.8 ^c^	24.8 ± 0.2 ^b^	5.49 ± 0.18 ^a^	1.60 ± 0.09 ^a^
OMSF (control)	85 ± 11 ^CD^	31.3 ± 1.9 ^A^	24.4 ± 1.4 ^C^	5.39 ± 0.96 ^C^	1.41 ± 0.08 ^B^
OMSF+MH	83 ± 8 ^D^	25.1 ± 1.2 ^D^	26.9 ± 1.8 ^B^	5.52 ± 0.06 ^C^	1.57 ± 0.09 ^B^
OMSF+BH	92 ± 6 ^BC^	28.7 ± 0.9 ^BC^	20.4 ± 1.2 ^E^	5.33 ± 0.45 ^D^	1.54 ± 0.09 ^B^
OMSF+BP	86 ± 5 ^CD^	30.8 ± 0.3 ^AB^	8.23 ± 0.2 ^F^	5.91 ± 0.25 ^C^	1.43 ± 0.08 ^B^
OMSF+BB	94 ± 5 ^B^	30.3 ± 1.1 ^AB^	22.8 ± 0.8 ^D^	8.13 ± 0.45 ^B^	1.56 ± 0.09 ^B^
OMSF+P	115 ± 11 ^A^	27.5 ± 1.1 ^C^	39.1 ± 1.4 ^A^	9.27 ± 0.29 ^A^	1.91 ± 0.11 ^A^
LSD_0.05_	6.9	1.94	2.02	0.43	0.151

^1^ Within columns, values subscribed by the same small or capital letters do not differ significantly at *p* < 0.05. Data are means of at least three determinations. LSD_0.05_—least significant difference at significance level 0.05 calculated within column for group of all samples. ^2^ Abbreviations of starch films: NMSF—native maize starch-based films, OMSF—octenyl-succinylated maize starch-based films, MH—multiflower honey, BH—buckwheat honey, BP—bee pollen, BB—bee bread, P—propolis, WVP—water vapor permeability.

**Table 3:** Mechanical and thermal properties of native and OSA maize starch films without and HBE.

Film Samples ^2^	TS ^1^ [MPa]	%EB [%]	T_o_ (°C)	T_p_ (°C)	T_e_ (°C)	ΔH (J/g)
NMSF (control)	8.3 ± 1.4 ^a^	37.0 ± 4.2 ^d^	269.7 ± 4.9 ^a^	271.4 ± 4.0 ^a^	275.7 ± 3.9 ^a^	102.0 ± 2.8 ^a^
NMSF+MH	6.4 ± 0.4 ^b^	56.8 ± 7.4 ^c^	248.7 ± 2.9 ^b^	250.0 ± 3.1 ^b^	255.2 ± 2.6 ^b^	87.4 ± 2.8 ^b^
NMSF+BH	6.9 ± 0.6 ^b^	55.0 ± 6.7 ^c^	254.4 ± 5.6 ^b^	256.3 ± 5.5 ^b^	260.4 ± 6.4 ^b^	86.6 ± 3.3 ^b^
NMSF+BP	6.1 ± 0.5 ^b^	74.6 ± 7.7 ^a^	247.1 ± 1.6 ^b^	249.2 ± 1.7 ^b^	254.7 ± 1.3 ^b^	83.0 ± 2.9 ^b^
NMSF+BB	4.2 ± 0.5 ^c^	66.2 ± 7.7 ^b^	252.0 ± 6.1 ^b^	254.5 ± 6.1 ^b^	261.2 ± 6.0 ^b^	88.9 ± 0.7 ^b^
NMSF+P	3.5 ± 0.3 ^d^	44.6 ± 6.8 ^d^	249.8 ± 5.7 ^b^	252.0 ± 6.2 ^b^	257.7 ± 6.9 ^b^	69.8 ± 6.3 ^c^
OMSF (control)	4.0 ± 0.4 ^B^	40.1 ± 6.7 ^D^	248.0 ± 4.0 ^A^	250.8 ± 4.1 ^A^	257.4 ± 3.6 ^A^	70.5 ± 1.0 ^AB^
OMSF+MH	3.5 ± 0.3 ^C^	67.7 ± 7.6 ^B^	232.3 ± 1.8 ^BC^	234.3 ± 1.7 ^BC^	239.6 ± 1.1 ^BC^	68.4 ± 1.3 ^BC^
OMSF+BH	4.5 ± 0.3 ^A^	70.4 ± 7.9 ^AB^	218.4 ± 8.8 ^D^	220.0 ± 8.7 ^CD^	224.9 ± 8.8 ^D^	67.5 ± 3.7 ^BC^
OMSF+BP	2.9 ± 0.3 ^D^	77.5 ± 7.5 ^A^	240.8 ± 3.0 ^AB^	243.1 ± 2.8 ^AB^	248.1 ± 3.7 ^AB^	75.0 ± 2.9 ^A^
OMSF+BB	2.4 ± 0.4 ^E^	70.4 ± 6.7 ^A^	216.4 ± 1.9 ^D^	218.0 ± 1.7 ^D^	223.6 ± 2.7 ^D^	55.3 ± 5.5 ^D^
OMSF+P	1.4 ± 0.1 ^F^	58.5 ± 6.5 ^C^	226.1 ± 12.1 ^CD^	228.0 ± 11.8 ^CD^	233.6 ± 12.2 ^C^	63.9 ± 2.8 ^C^
LSD_0.05_	0.64	8.0	9.71	9.45	9.82	5.6

^1^ Within columns, values subscribed by the same small or capital letters do not differ significantly at *p* < 0.05. Data are means of at least three determinations. LSD_0.05_—least significant difference at significance level 0.05 calculated within column for group of all samples. ^2^ Abbreviations of starch films: NMSF—native maize starch film, OMSF—OSA maize starch films, MH—multifloral honey extract, BH—buckwheat honey extract, BP—bee pollen extract, BB—bee bread extract, P—propolis extract, TS—tensile strength, %EB—percent elongation at break, T_0_—onset temperature, T_p_—peak temperature, T_e_—end temperature, ΔH—enthalpy change.

**Table 4:** Total phenolic content, antioxidant activity, and phenolic profile of native and octenyl-succinylated maize starch-based films with HBE.

Film Sample ^2^	Antioxidant Activity		
	TPC ^1^ [mg GAE/g of Film]	DPPH [mg TE/g of Film]	ABTS [mg TE/g of Film]
NMSF (control)	0.02 ± 0.00 ^f^	0.06 ± 0.01 ^d^	0.62 ± 0.03 ^f^
NMSF+MH	0.09 ± 0.00 ^e^	0.08 ± 0.02 ^d^	0.79 ± 0.02 ^e^
NMSF+BH	0.19 ± 0.01 ^d^	0.09 ± 0.02 ^d^	1.39 ± 0.06 ^d^
NMSF+BP	0.54 ± 0.01 ^c^	0.19 ± 0.08 ^c^	2.90 ± 0.02 ^c^
NMSF+BB	0.62 ± 0.04 ^b^	0.29 ± 0.02 ^b^	3.17 ± 0.01 ^b^
NMSF+P	27.10 ± 0.10 ^a^	1.77 ± 0.10 ^a^	22.13 ± 0.46 ^a^
OMSF (control)	0.08 ± 0.01 ^E^	0.07 ± 0.03 ^E^	0.54 ± 0.05 ^F^
OMSF+MH	0.14 ± 0.01 ^D^	0.12 ± 0.04 ^D^	0.75 ± 0.01 ^E^
OMSF+BH	0.17 ± 0.00 ^D^	0.12 ± 0.01 ^D^	2.32 ± 0.02 ^D^
OMSF+BP	1.23 ± 0.00 ^C^	0.58 ± 0.01 ^C^	5.96 ± 0.03 ^C^
OMSF+BB	1.32 ± 0.05 ^B^	0.63 ± 0.02 ^B^	6.52 ± 0.04 ^B^
OMSF+P	28.28 ± 0.35 ^A^	2.20 ± 0.15 ^A^	24.59 ± 1.21 ^A^
LSD_0.05_	0.76	0.10	0.63

^1^ Within columns, values subscribed by the same small or capital letters do not differ significantly at *p* < 0.05. Data are means of at least three determinations. LSD_0.05_—least significant difference at significance level 0.05 calculated within column for group of all samples. ^2^ Abbreviations of film samples: NMSF—native maize starch-based films, OMSF—ocetynyl-succinylated maize starch-based films, MH—multifloral honey, BH—buckwheat honey, BP—bee pollen, BB—bee bread, P—propolis.

**Table 5:** Effects of HBE incorporation on the inhibition zone of native and octenyl-succinylated maize starch-based films against Gram-positive and Gram-negative bacteria.

Sample Name ^2^	Inhibition Zone [mm]											
	St1 ^1^	St2	St3	St4	St5	St6	St7	St8	St9	Bs	Sal	Ec
NMSF	0	0	0	0	0	0	0	0	0	0	0	0
NMSF+MH	0	0	0	0	0	0	0	0	0	0	0	0
NMSF+BH	0	0	0	0	0	0	0	0	0	0	0	0
NMSF+BP	9.5 ± 1.0	9.5 ± 1.0	0	0	10.0 ± 1.2	0	0	0	10.0 ± 1.2	0	0	0
NMSF+BB	0	13.5 ± 2.5	0	0	12.5 ± 1.9	0	0	0	0	25.0 ± 0.0	0	11.0 ± 0.0
NMSF+P	0	11.0 ± 1.6	15.5 ± 1.0	13.0 ± 1.6	0	13.5 ± 1.0	0	21.0 ± 0.0	13.5 ± 1.9	11.0 ± 0.0	15.0 ± 0.0	17.0 ± 5.4
OMSF	0	0	0	0	0	0	0	0	0	0	0	0
OMSF+MH	0	0	0	0	0	0	0	0	0	0	0	0
OMSF+BH	0	0	0	0	7.3 ± 4.9	0	0	0	0	0	0	0
OMSF+BP	10.5 ± 1.9	12.0 ± 2.6	0	0	11.5 ± 1.0	0	0	4.5 ± 5.2	0	0	0	0
OMSF+BB	0	11.0 ± 1.6	0	0	8.3 ± 5.7	0	0	11.0 ± 1.6	0	11.0 ± 0.0	0	25.0 ± 0.0
OMSF+P	0	9.5 ± 1.0	13.0 ± 0.0	21.5 ± 2.5	0	10.5 ± 1.9	0	23.0 ± 3.7	16.0 ± 2.6	15.0 ± 2.8	25.0 ± 0.0	18.8 ± 6.1

^1^ Within columns, the means and standard deviations are calculated based on four determinations. ^2^ Abbreviations: NMSF—native maize starch-based films, OMSF—ocetynyl-succinylated maize starch-based films, MH—multiflower honey, BH—buckwheat honey, BP—bee pollen, BB—bee bread, P—propolis.

**Table 6:** Quality parameters of apple slices before and after 7-day storage in films based on native and octenyl-succinylated maize starch-based films with HBE.

Name of Sample ^2^	Lightness ^1^ L* [-]	Browning Index BI [-]	Water Activity (aw) [-]	Weight Loss [%]
fresh apple	79.9 ± 1.1 ^a^	46.0 ± 3.6 ^d^	0.854 ± 0.001 ^a^	-
After 7 days of storage				
apple in NMSF apple in NMSF+MH apple in NMSF+BH apple in NMSF+BP apple in NMSF+BB apple in NMSF+P	42.2 ± 4.6 ^de^ 40.9 ± 3.8 ^def^ 37.4 ± 1.5 ^f^ 43.1 ± 1.7 ^de^ 40.8±4.1 ^def^ 47.8±1.5 ^c^	112.3 ± 6.5 ^a^ 108.6 ± 8.8 ^a^ 109.3 ± 6.3 ^a^ 115.2 ± 4.7 ^a^ 119.4 ± 5.4 ^a^ 120.0± 12.5 ^a^	0.732± 0.008 ^bc^ 0.758 ± 0.011 ^b^ 0.715 ± 0.008b ^cd^ 0.736 ± 0.001 ^bc^ 0.732 ± 1.13 ^bc^ 0.697 ± 0.040 ^cd^	59.48 ± 5.37 ^ab^ 55.48 ± 4.09 ^c^ 60.14 ± 1.55 ^abc^ 56.13 ± 2.99 ^c^ 56.45 ± 4.96 ^bc^ 58.74 ± 1.14 ^abc^
apple in OMSF apple in OMSF+MH apple in OMSF+BH apple in OMSF+BP apple in OMSF+BB apple in OMSF+P	44.9 ± 3.7 ^cd^ 43.5 ± 2.0 ^cde^ 40.6 ± 3.6 ^def^ 40.8 ± 4.1 ^def^ 44.7 ± 3.5 ^cd^ 42.1 ± 3.5 ^de^	105.9 ± 5.9 ^b^ 107.8 ± 11.7 ^b^ 103.3 ± 11.3 ^b^ 105.7 ± 10.1 ^b^ 122.6 ± 8.0 ^a^ 120.0 ± 11.9 ^a^	0.719 ± 0.004b ^cd^ 0.695 ± 0.009 ^c^ 0.683 ± 0.013 ^d^ 0.683 ± 0.003 ^d^ 0.697 ± 0.026 ^cd^ 0.634 ± 0.055 ^e^	60.59 ± 1.91 ^ab^ 59.67 ± 2.18 ^abc^ 59.19 ± 1.25 ^abc^ 61.88 ± 2.27 ^a^ 61.02 ± 1.75 ^ab^ 63.04 ± 0.44 ^a^
apple in LDPEF	52.3 ± 4.6 ^b^	76.0 ± 7.3 ^c^	0.850 ± 0.0115 ^a^	2.37 ± 0.35 ^a^
LSD0.05	4.34	11.66	0.045	4.69

^1^ Within columns, values subscribed by the same letters do not differ significantly at *p* < 0.05. ^2^ Abbreviations: NMSF—native maize starch-based films, OMSF—ocetynyl-succinylated maize starch-based films, MH—multiflower honey, BH—buckwheat honey, BP—bee pollen, BB—bee bread, P—propolis, LDPEF—low-density polyethylene film.

## Data Availability

Data are contained within the article.
